# Luncheon to the Uniformed Auxiliary Services

**Published:** 1919-11-01

**Authors:** 


### Luncheon to the Uniformed Auxiliary Services.

A VERY enjoyable luncheon was given on Wednesday, October 22, at the Trocadero Restaurant by a number of representative women to the heads of the Uniformed Auxiliary Services. The guests were Lady Ampthill, representing the V.A.D.s; Lady Londonderry, the Women's Legion; Dame Florence Burleigh Leach, Q.M.A.A.C.; Dame Katharine Furse, W.R.N.S. (who was unavoidably absent); Mrs. Gwynne Vaughan, W.R.A.F.; Miss Meriel Talbot, the Land Army; and Dr. Flora Murray, Endell Street Hospital. Dame Sidney Browne was also among the company, who were restricted to one hundred. Princess Alice, Countess of Athlone, honoured the party with her presence, and the Queen most graciously sent flowers for the tables. Miss M. F. Billington acted as secretary, and Mrs. Massey Lyon as chairman. Dr. Mary Scharlieb was also a member of the committee, as was Lady Bland Sutton, who kindly lent her house for its meeting, and Mrs. Garrett Anderson.

A beautiful little address of welcome to the guests, written by Mrs. Meynell, was read by Miss Lena Ashwell. "Voluntary self-sacrifice," Mrs. Meynell said, "is the greatest thing in all the world. It is the redemption of all things. It has redeemed us. It has redeemed the most detestable thing on earth—war itself. When I sailed into New York harbour and saw the statue of Liberty, I wished it might be renamed the statue of Voluntary Obedience, for that is surely the form of liberty that is the one great antithesis of slavery. Little, at the outset, was asked or expected of women in the war except endurance. They were, therefore, the freest, even from the constraint or the suggestion of public opinion. Many women did not merely accept the duty of public service, they assumed it. But even when the stress and necessity and obligation came into force the nation never lost the virtue of the voluntary worker, the voluntary soldier. For in entering the war *the nation itself, the State, the people, had volunteered.*"

Lady Ampthill returned thanks in an admirable little speech.

